# Matrix-Assisted Laser Desorption/Ionization Imaging Mass Spectrometry

**DOI:** 10.3390/ijms11125040

**Published:** 2010-12-07

**Authors:** Nobuhiro Zaima, Takahiro Hayasaka, Naoko Goto-Inoue, Mitsutoshi Setou

**Affiliations:** Department of Molecular Anatomy, Hamamatsu University School of Medicine, 1-20-1 Handayama, Higashi-ku, Hamamatsu, Shizuoka 431-3192, Japan; E-Mails: thaya@hama-med.ac.jp (T.H.); naoko.goto.inoue@gmail.com (N.G.-I.); setou@hama-med.ac.jp (M.S.)

**Keywords:** imaging mass spectrometry, matrix-assisted laser desorption/ionization mass spectrometry, biomarker, protein, lipids

## Abstract

Matrix-assisted laser desorption/ionization mass spectrometry (MALDI-MS) is a powerful tool that enables the simultaneous detection and identification of biomolecules in analytes. MALDI-imaging mass spectrometry (MALDI-IMS) is a two-dimensional MALDI-mass spectrometric technique used to visualize the spatial distribution of biomolecules without extraction, purification, separation, or labeling of biological samples. MALDI-IMS has revealed the characteristic distribution of several biomolecules, including proteins, peptides, amino acids, lipids, carbohydrates, and nucleotides, in various tissues. The versatility of MALDI-IMS has opened a new frontier in several fields such as medicine, agriculture, biology, pharmacology, and pathology. MALDI-IMS has a great potential for discovery of unknown biomarkers. In this review, we describe the methodology and applications of MALDI-IMS for biological samples.

## 1. Overview

**Figure 1.**
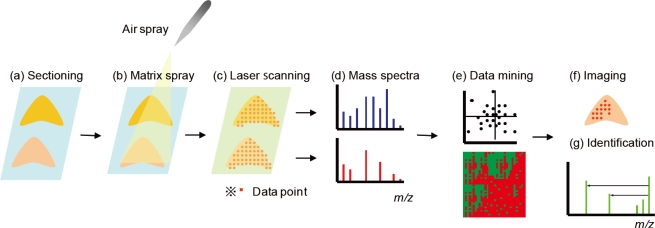
Schema of biomarker discovery using MALDI-IMS. Thin-section samples are mounted on a plate (**a**), coated with matrix (**b**), and measured by a mass spectrometer (**c**). Obtained mass spectra (**d**) are used for a data mining approach (**e**). Molecules of interest can be visualized (**f**) and identified by MS/MS of tissues (**g**).

Imaging mass spectrometry (IMS) is a relatively new imaging method based on mass spectrometry (MS). MS is an analytical technique that detects the mass-to-charge ratio (*m/z*) of ionized molecules. The application of several ionization methods, including secondary ion mass spectrometry (SIMS) [1], desorption electrospray ionization (DESI) [2], and matrix-assisted laser desorption/ionization (MALDI) [3], has been investigated for IMS. MALDI-MS is a powerful method that allows the simultaneous detection and identification of many molecules directly from biological sections [4,5]. MALDI-MS can detect a wide range of biomolecules ranging from small (*m/z* < 1000) [6] to large molecules (*m/z* > 100 kDa) [7]. Due to the widespread applicability of this method, MALDI-MS is widely used in various fields, such as proteomics [8–10], lipidomics [11–18], metabolomics [19,20], and glycomics [21,22]. In this review, we focus on molecular imaging by MALDI-MS (MALDI-IMS), a two-dimensional MALDI-MS technique that is used to visualize the spatial distribution of biomolecules in biological sections (Figure 1) [23–30]. Samples for MALDI-IMS can be obtained from any type of organism, ranging from bacteria and plants to animal and human tissues. The versatility of MALDI-IMS has opened a new frontier in several fields, including medicine, agriculture, biology, pharmacology, and pathology.

The important features that make MALDI-IMS a practical tool in a wide range of life sciences are its molecular identification capability and label-free biomolecular simultaneous imaging. These features of MALDI-IMS provide the scientific community with a new strategy for biomarker discovery. This new non-targeting screening method has proven useful for discovering unknown biomarkers [31–33]. In this review, we describe the methodology and applications of MALDI-IMS for biological samples.

## 2. Methodology

The important experimental steps for visualizing endogenous molecules by using MALDI-IMS are sample preparation, matrix application, measurement, and data analysis. In this section, we describe the basic experimental MALDI-IMS procedure.

### 2.1. Biological Sample Preparation

Optimization of the sample preparation procedure according to the characteristic chemical and physical properties of each analyte is important. The sample preparation procedure needs to be controlled to obtain meaningful images of endogenous molecules in the microdomain of biological sections. Here, we discuss crucial preparation steps, including sample fixation, embedding, sectioning, and washing.

#### 2.1.1. Fixation and Embedding

The most preferred samples for MALDI-IMS are fresh or frozen and chemically unmodified tissues. Fresh-frozen tissues can be prepared using powdered dry ice, liquid nitrogen, liquid nitrogen-chilled isopentane, *etc*. Among these fresh-freezing methods, the tissue section morphology appears to be well-maintained when samples are frozen by liquid nitrogen-chilled isopentane, which is used to freeze biopsy samples in clinical practice [34]. However, the optimal freezing method differs for different samples. It is important to ensure that tissue section morphology is well maintained.

Embedding of the tissue samples in supporting material, such as an optimal cutting temperature (OCT) compound, allows maintenance of tissue morphology and precise sample sectioning. However, supporting materials are often ionized during MALDI-MS analysis and sometimes act as ion suppressors of molecules of interest [35]. Therefore, samples should not be embedded if precise sample sections can be prepared without embedding. When it is difficult to prepare a sample section, the use of carboxymethylcellulose (CMC) or gelatin as embedding material is recommended. Sodium CMC (2%) is reported to be used as an alternative embedding compound that does not interfere with the detection sensitivity of biomolecules in MALDI-IMS analysis [36,37]. Chen *et al*. reported that gelatin provided a cleaner signal background compared to that provided by OCT [38]. Researchers should ensure suitability between the supporting material and the biomolecules of interest.

Formaldehyde fixation and paraffin embedding, the most commonly used preservation technique, has limited use in IMS. Formaldehyde fixation makes IMS analysis difficult because of the protein cross-linking induced by formaldehyde. The on-tissue proteolytic digestion method, in which proteins are denatured and digested by enzymes, has been developed in order to address this problem [9,39]. The protocol for this approach was recently published [40], and the method includes an indispensable paraffin removal step involving the use of xylene and ethanol. Lipophilic molecules are lost during this deparaffinization step; therefore, formaldehyde-fixed and paraffin-embedded (FFPE) samples cannot be used for lipid imaging.

#### 2.1.2. Sectioning

Ionization efficiency is partly dependent on the thickness of the tissue section [41]. In general, 5–20-μm-thick sections are prepared for the analysis of low molecular weight molecules. The use of thinner tissue sections (2–5 μm thick) has been recommended for the analysis of high molecular weight molecules (range, 3–21 kDa) [42]. Sections are thaw-mounted on a stainless steel conductive stage or on commercially available indium-tin oxide (ITO)-coated glass slides. We recommend the use of ITO-coated glass slides because these transparent slides enable microscopic observation of the section after MALDI-IMS. Adhesive film can be used instead of thaw-mounting on the ITO-coated glass slides [36]. Use of adhesive film is suitable for samples for which thaw-mounted preparation of sections is challenging (e.g., rice kernel, bone, or whole-body sections) [43].

#### 2.1.3. Washing

Washing is required for peptide or protein analysis because their detection is often prevented by large amounts of easily ionized lipid species. Lipid removal simplifies mass spectra in the range of *m/z* 400–1000; thus, lipid removal enables the detection of low mass peptides that are usually masked by lipid peaks. The washing method should be optimized for the target imaging molecules. Several washing protocols using organic solvents have been reported [9,35,44–46]. Washing is also required after MALDI-IMS for removing the staining matrix. The matrix can be removed by the solvent after IMS. For example, 2,5-dihydroxybenzoic acid (DHB) can be removed by methanol. After MALDI-IMS completion and matrix removal, tissue samples can be stained with hematoxylin-eosin.

### 2.2. Matrix Application

The matrix plays a central role in MALDI-MS soft ionization [4,5]. Biomolecules are softly ionized in the co-crystal with the matrix, which absorbs the laser beam energy and protects biomolecules from the disruptive energy. Protonated ion ([M + H]^+^) or deprotonated ion ([M − H]^−^) molecules are generally detected. Sodium adduct ion ([M + Na]^+^) and potassium adduct ion ([M + K]^+^) are often observed by biological sample analysis. It is very important to choose appropriate matrices to obtain meaningful biomolecule images. An overview of the matrices used for IMS can also be found in other reviews [30,47].

#### 2.2.1. Choice of Matrix

The choice of matrix used for MALDI-IMS depends on the mass range and chemical properties of the analytes. Among the many kinds of matrices, sinapinic acid (3,5-dimethoxy-4-hydroxycinnamic acid [SA]) is generally used for high-weight molecules such as proteins, while α-cyano-4-hydroxycinnamic acid (CHCA) is often used for middle-weight molecules such as peptides. DHB or 9-aminoacridine (9-AA) is generally used for low molecular weight molecules such as lipids or metabolites. DHB appears to be suitable for analysis in the positive-ion mode, while 9-AA tends to be appropriate for analysis in the negative-ion mode.

The development of new matrices is still being reported. Ours and other research groups recently reported the use of nanoparticles as new matrices [48–52]. For example, iron oxide nanoparticles enable the visualization of sulfatide and phospholipid distribution [22,53], silver nanoparticles can be used for the analysis of fatty acids [50], and gold nanoparticles are appropriate for the sensitive detection of glycosphingolipids such as sulfatide and ganglioside [54].

#### 2.2.2. Matrix Application Methods

Three kinds of matrix application methods are generally used. The first method involves manual spraying using thin layer chromatography (TLC) sprayers or airbrushes [55]. This method should be performed at a constant room temperature and at low humidity to ensure homogeneous application of the matrix. By applying this method, tissue sections can be quickly and inexpensively coated with relatively small crystals. However, this method requires skill. The second method is automatic depositing of small droplets of matrix solution with robotic devices such as a chemical inkjet printer (ChIP-1000) (Shimadzu Corporation, Kyoto, Japan) [56]. The on-tissue digestion method mentioned above can also be performed using the ChIP-1000 instrument. Compared to the droplet spot size in manual spraying, the droplet spot size of this method increases the signal sensitivity but decreases the spatial resolution (>100–200 μm) [57]. The limitation of the inkjet printer is capillary clogging, which occurs when highly concentrated matrix solutions are used. The third method is sublimation of the matrix under reduced pressure and elevated temperature [58]. This method requires no solvent; therefore, diffusion of the analyte molecules during matrix application is eliminated. Another advantage is the increased purity of the matrix and the formation of very small matrix crystals [59].

### 2.3. Measurement and Data Analysis

#### 2.3.1. Instruments

The requirement for performance of IMS is the availability of an *x-axis-y-axis* moving stage with electronic controls. Most modern MS instruments produced by major MS hardware companies (*i.e.*, Shimadzu, ThermoFisher Scientific, Bruker, Applied Biosystems, Waters) can be adapted for MALDI-IMS. Time of flight (TOF) is the most widely used technology. TOF analyzers allow the separation of ionized accelerated molecules according to their mass to charge (*m/z*) ratio. TOF-MS offers suitable performance for MALDI-IMS, namely, good transmission ratio (50–100%), sensitivity, mass range, and repetition rate. However, TOF-MS lacks the capability to perform effective tandem MS experiments. This disadvantage of TOF-MS changed with the introduction of hybrid analyzers such as combination of quadrupole mass analyzer and TOF (so-called qTOF), combination quadrupole ion trap (QIT) and TOF (so-called QIT-TOF), combination of ion mobility spectrometry (IMS) and TOF (so-called IMS-TOF), or combination of two TOF mass spectrometers (so-called TOF-TOF). These combination systems revolutionized the application of TOF-MS systems for the structural analysis with tandem MS. In general, the first system is used to select a precursor ion for fragmentation, while the second TOF system is employed for fragment analysis. Other mass analyzers (and their combinations), such as linear ion trap (LIT) [36,60,61], triple quadrupole (QqQ) [62], and Fourier transform ion cyclotron resonance (FTICR) [63], are used for MALDI-IMS. The advantages of commercially available LIT instruments are miniaturization, capability of sample analysis on nonconductive glass slide MALDI performance at intermediate pressure, and superior performance of multistage MS. The QqQ system allows quantitative analysis and single or multiple reaction monitoring (SRM/MRM). The FTICR system offers very high mass resolving power and high mass measurement accuracy.

#### 2.3.2. Measurement

Matrix-coated samples should be measured as soon as possible to avoid biomolecule degradation. The procedure to obtain a good MALDI-IMS spectrum is basically the same as that of traditional MALDI-MS. Since MALDI-IMS is a two-dimensional MALDI-MS technique, the measurement region and scan pitch, which decide the spatial resolution of the image, need to be fixed before the measurement is taken. At present, the finest spatial distribution of the commercially available instrument is 10 μm (Mass Microscope Shimadzu) [64,65]. The measurement time depends on the number of data spots, the frequency of the laser, the number of shots per spot, and the time required to move the sample stage. For example, when researchers select the region of interest as a 1 × 1 mm^2^ area with a 10-μm scan pitch (10,000 data points), it takes about 1 h to complete the measurement using a mass microscope equipped with a 1000-Hz laser (100 shots/data point).

#### 2.3.3. Data Analysis

A large amount of data (a few gigabytes) is obtained by MALDI-IMS; therefore, visualization software packages that can rapidly and efficiently analyze enormous spectra have been developed. BioMap (a free software; Novartis, Basel, Switzerland), FlexImaging (Bruker Daltonics, Bremen, Germany), and ImageQuest (Thermo Fisher Scientific, CA, USA) are generally used for visualization. For biomarker analysis of the MALDI-IMS dataset, data mining software such as ClinProTools (Bruker Daltonics) are typically used [66]. These data mining software effectively reduce the number of biomarker candidates. We previously reported the approach using principal component analysis (PCA) to discover different biomolecules between starvation-induced fatty livers and normal livers [55]. Hierarchical clustering was also used to analyze the data obtained from gastric cancer and non-neoplastic mucosa tissue sections [67].

## 3. Applications of IMS

### 3.1. IMS for Proteins and Peptides

The proteins present in biological tissue are usually subjected to on-tissue digestion by proteolytic enzyme such as trypsin. Groseclose *et al*. reported the protocol for protein imaging [9]. The method for on-tissue protein digestion should include a tissue washing step with organic solvent for removing lipids. After lipid removal, a tissue section is spotted with the solution of proteolytic enzyme. Use of a chemical inkjet printer (such as ChIP-1000) is suitable for the application of proteolytic enzymes. One of the advantages of IMS is its label-free simultaneous visualization of biomolecules. In protein or peptide studies, the advantage of MALDI-IMS is utilized when analytes have variants or when non-targeting screening is performed. Stoeckli *et al*. visualized the amyloid-β peptide species, which is generated by cleaving the amyloid precursor protein at different cleavage sites [68]. Yao *et al*. visualized the biomarkers of the neurodegenerative disease model mouse (Scrapper-knockout mouse) [69] using PCA [8]. Applications of MALDI-IMS have been reported in cancer pathology, such as human gastric cancer tissues using FFPE samples [56]. MALDI-IMS has been applied in a vast spectrum of disease samples and models [39,70–74].

Non-digestion approaches have also been reported. This approach was first reported by Caprioli *et al*. [25]. One of the interesting applications for intact proteins directly from tissue sections was reported by Dani *et al*. [10]. They succeeded in the identification of two proteins: an 8 kDa protein that matched an unannotated sequence of the *Anopheles gambiae* genome and odorant binding protein 9 (OBP-9). A comparison of the peptide profiles of the neurons of the sea slug (*Aplysia californica*) was reported by Rubakhin *et al*. [75], who revealed distinct peptide profiles for each neuronal subtype.

### 3.2. IMS for Lipids

**Figure 2.**
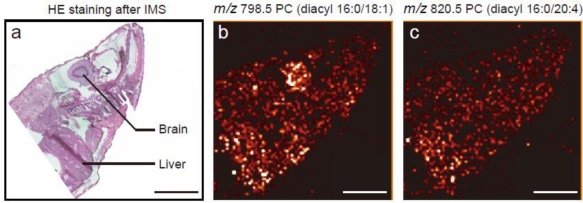
Ion images of phosphatidylcholine (PC) in medaka (*Oryzias latipes*). Scale bar: 2 mm. (**a**) Hematoxylin-eosin staining after MALDI-IMS. (**b**) Distribution of PC (diacyl 16:0/18:1). (**c**) Distribution of PC (diacyl 16:0/20:4).

**Figure 3.**
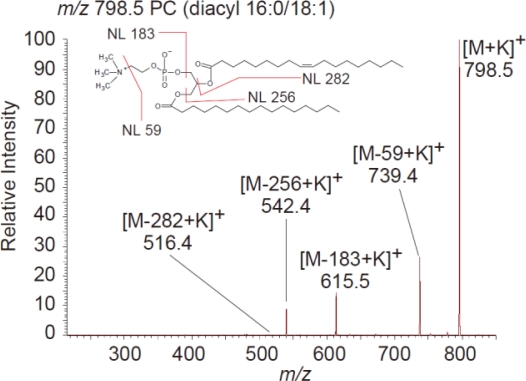
Phosphatidylcholine (PC) MS/MS spectrum at *m/z* 798.5. Neutral loss of the PC head group and fatty acids is generally observed by MS/MS analysis of phospholipids in tissues.

IMS is currently the only methodology that enables simultaneous visualization of lipids. Visualization of various types of lipids, such as phospholipids [23,27,76–79], sphingolipids [28], glycolipids [13,21,80], and neutral lipids [81], has been reported. In terms of tissue sample preparation, formaldehyde fixation and OCT embedding are not recommended for lipid imaging because of a significant reduction in the quality of the mass spectra. Figure 2 shows an example of lipid imaging in a biological sample, medaka (*Oryzias latipes*) [23]. IMS reveals the distribution of lipids in biological tissues at the molecular species level. Phosphatidylcholine (PC) (diacyl 16:0/18:1) was observed to be localized in the brain and liver, while PC (diacyl 16:0/20:4) was intensely detected in the liver (Figures 2a and 2b). Molecular lipid species can be identified by MS/MS tissue analyses. A representative MS/MS PC spectrum is shown in Figure 3. Neutral loss of the phosphocholine head group (59 and 183 Da) and fatty acids are usually observed by MS/MS analyses of PC in tissues. Other phospholipid species commonly exhibit neutral loss of the head group (43 Da for phosphatidylethanolamine, 162 Da for phosphatidylinositol, 87 Da for phosphatidylserine, and 59 and 183 Da for sphingomyelin) on MS/MS spectra [82]. Neutral loss of fatty acids is observed in the case of MS/MS analyses for triacylglycerol [81].

We recently reported the unique distribution of PC in varicose veins (VVs) [83]. In this study, we found arachidonic acid containing PC was localized in the damaged valvular region of VVs, suggesting that local inflammation is initiated by the arachidonic acid concentration via an unknown mechanism. We further observed this unique lipid distribution pattern in other vascular diseases. Because lipids are strongly involved in the onset of vascular diseases [84], abnormal localization of lipids might be a key clue in clarifying the unknown mechanism of vascular disease onset. The use of adhesive film is appropriate for arteriosclerotic arteries that have massive calcified regions [36,43].

### 3.3. IMS for Endogenous Metabolites

MALDI-IMS has been employed for the detection of endogenous molecules such as adenosine monophosphate (AMP), adenosine diphosphate (ADP), adenosine triphosphate (ATP), and uridine diphosphate (UDP) using 9-AA [85]. Hattori *et al*. measured the change of ATP, ADP, and AMP in an ischemic brain model [86].

**Figure 4.**
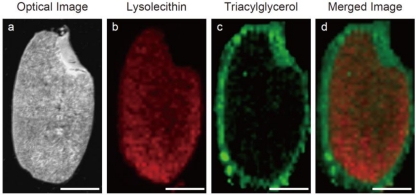
Ion image of nutrients in rice (*Oryza sativa*). Scale bar: 1 mm. (**a**) Optical image of a rice kernel (*Hinohikari*). (**b**) Distribution of lysolecithin. (**c**) Distribution of triacylglycerol. (**d**) Merged image of lysolecithin (red) and triacylglycerol (green). The rice kernel section can be prepared using adhesive film.

Food nutrients can be visualized by IMS [20,36,82,87,88]. We recently analyzed agricultural and marine products and visualized food nutrients such as γ-aminobutyric acid (GABA), essential amino acids, sucrose, α-tocopherol, γ-oryzanol, and phytic acid [36,82,87]. Volatile components can be visualized by a mass microscope equipped with an atmospheric pressure ion-source chamber (Shimadzu) [64]. Figure 4 shows the food nutrients of rice (*Oryza sativa*) [36]. In this study, we found it possible that nutrient distributions are different among rice varieties. The distribution of nutrients might be used as a biomarker to discriminate the geographical origin of agricultural and marine products. A larger-scale study is required to confirm our findings.

## 4. Future Perspectives

MALDI-IMS is becoming an essential tool for molecular imaging of biological samples. MALDI-IMS can facilitate the discovery of characteristic molecules in regions of interest. The advantage of MALDI-IMS—in that it enables us to investigate the localization of known and unknown molecules without labeling—should facilitate biomarker discovery and validation. To make the relatively new MALDI-IMS strategy a routine tool for biomarker discovery, it must first be validated in a larger-scale sample. A combination of MALDI-IMS and other IMS such as SIMS [1], DESI [2], or laser ablation inductively coupled plasma mass spectrometry (LA-ICP-MS) [89] is also needed to overcome the MALDI-IMS limitations. SIMS utilizes a primary ion beam to produce secondary ions from the surface of the biological sections and is a superior tool for high spatial resolution IMS (submicron order) of elements and small molecules at the organelle level [90–92]. However, SIMS lacks the sensitivity of the mass range over *m/z* 1000 due to in-source fragmentation as well as molecular identification ability due to MS/MS incapability. We recently reported that combination use of MALDI-IMS and SIMS-IMS complements their respective limitations [93]. DESI is a combination of two MS ionization methods, namely electrospray ionization and desorption ionization. DESI uses energetic charged electrosprayed solvent droplets to desorb molecules from the sample surface. Although the spatial resolution of DESI-IMS is inferior to that of MALDI-IMS, DESI-IMS allows for soft and atmospheric desorption and ionization, which could overcome the MALDI-IMS limitation. DESI was applied in several studies including metabolites [94], alkaloids in plant [2], and exogenous and endogenous chemicals in latent fingerprints [95]. LA-ICP-MS has been developed as a method for imaging elements and is the most sensitive technique for elemental imaging of biological tissues [96]. Quantitative analysis can be performed by LA-ICP-MS. Becker *et al*. proposed the combined application of LA-ICP-MS and MALDI-MS [96]. We believe that MALDI-IMS or combination with alternative IMS will become an essential tool for biomarker discovery in the near future.
